# Efficient Constant Envelope Precoding for Massive MU-MIMO Downlink via Majorization-Minimization Method

**DOI:** 10.3390/e26040349

**Published:** 2024-04-21

**Authors:** Rui Liang, Hui Li, Yingli Dong, Guodong Xue

**Affiliations:** School of Electronics and Information, Northwestern Polytechnical University, Xi’an 710129, China; liangrui@mail.nwpu.edu.cn (R.L.); d.y.l@mail.nwpu.edu.cn (Y.D.); xgd520@mail.nwpu.edu.cn (G.X.)

**Keywords:** alternating minimization, constant envelope precoding, majorization-minimization method, fast iterative shrinkage-thresholding method, second-order Taylor expansion

## Abstract

The practical implementation of massive multi-user multi-input–multi-output (MU-MIMO) downlink communication systems power amplifiers that are energy efficient; otherwise, the power consumption of the base station (BS) will be prohibitive. Constant envelope (CE) precoding is gaining increasing interest for its capability to utilize low-cost, high-efficiency nonlinear radio frequency amplifiers. Our work focuses on the topic of CE precoding in massive MU-MIMO systems and presents an efficient CE precoding algorithm. This algorithm uses an alternating minimization (AltMin) framework to optimize the CE precoded signal and precoding factor, aiming to minimize the difference between the received signal and the transmit symbol. For the optimization of the CE precoded signal, we provide a powerful approach that integrates the majorization-minimization (MM) method and the fast iterative shrinkage-thresholding (FISTA) method. This algorithm combines the characteristics of the massive MU-MIMO channel with the second-order Taylor expansion to construct the surrogate function in the MM method, in which minimizing this surrogate function is the worst-case of the system. Specifically, we expand the suggested CE precoding algorithm to involve the discrete constant envelope (DCE) precoding case. In addition, we thoroughly examine the exact property, convergence, and computational complexity of the proposed algorithm. Simulation results demonstrate that the proposed CE precoding algorithm can achievean uncoded biterror rate (BER) performance gain of roughly 1dB compared to the existing CE precoding algorithm and has an acceptable computational complexity. This performance advantage also exists when it comes to DCE precoding.

## 1. Introduction

Massive MIMO technology is widely regarded as a revolutionary advancement in the fifth generation mobile communication system [1]. Compared with small-scale MIMO systems, large-scale antenna arrays at transmitters have been demonstrated to offer substantial benefits in terms of spectrum efficiency, energy efficiency, and reliable transmission [2,3,4]. In contrast to conventional small-scale MIMO systems that employ highly linear and power-inefficient radio frequency (RF) amplifiers, the practical implementation of massive MIMO systems requires the RF amplifiers to be power-efficient. Otherwise, the power consumption of the base station (BS) would be prohibitive. Therefore, it is crucial to use RF amplifiers with a certain power efficiency to avoid excessive power consumption at the BS [5]. Regrettably, energy-efficient RF amplifiers frequently exhibit inferior linearity characteristics, necessitating a lower peak-to-average power ratio (PAPR) for the input signals.

In response to the demand for energy-efficient and cost-effective RF components in wireless communication systems, constant envelope (CE) transmission that forces each antenna unit to transmit a constant envelope signal and allows the use of the most energy-efficient and cheapest power amplifiers (PAs) has attracted great attention from researchers. CE precoding was initially introduced by Mohammed and Larsson in [6]. Specifically, in the single-antenna CE constraint, the equivalent complex baseband signal of each transmit antenna is restricted to have a CE [7]. By combining with instantaneous CSI, CE precoding only transmits the phase of the desired information symbol to multiple antennas. CE precoding reduces the PAPR compared to non-CE precoding by providing a quasi-CE continuous-time RF signal to each PA. On the other hand, when highly efficient PAs with nonlinear amplitude transfer functions are used, the output distortion can be greatly reduced [8]. Furthermore, the low PAPR properties of the CE signals enable the utilization of cost-effective RF components that have a decreased dynamic range.

Based on all the advantages of CE precoding mentioned above, CE precoding has become a potential research direction. However, it is important to mention that the CE characteristics of the transmit signal are typically non-convex, which brings challenges to precoding design. By adopting multi-user interference (MUI) as the design goal, the CE precoding problem can be modeled as a non-convex nonlinear least squares (NLS) problem, and its local minimum can be obtained using the gradient descent method [9]. Nevertheless, the CE constraint can lead to the error-floor issue in situations with a high signal-to-noise-ratio (SNR). On this basis, the cross-entropy optimization (CEO) method was proposed in [10] to improve the CE precoding design optimal search. Furthermore, an alternating minimization projected gradient (GP-AltMin) method was proposed in [11]. This method improves the performance at high SNR by ignoring the noise of the system. Zhang et al. [12] studied a single-user multiple-input single-output (MISO) system with CE constraint for each antenna and proposed constellation designs for fixed-rate and variable-rate adaptive receivers. In [8], the joint design of transmit antenna grouping and receive beamforming vectors was conducted with the objective of minimizing the maximum symbol error rate (SER) in the data stream. Nevertheless, this typically necessitates a significant computational expenditure. Shao et al. [13] introduced a novel first-order algorithm that utilizes a projected gradient (PG) method in order to effectively minimize the SER of the system. To further accelerate the convergence rate of the PG method, a low-complexity fast gradient projection (FPG) algorithm similar to the fast iterative shrinkage-thresholding (FISTA) algorithm was considered. However, this approach necessitates a greater quantity of iterations in order to attain convergence. In [14], a novel algorithm was provided that integrates gradient extrapolation with the majorization-minimization technique (GEMM). This algorithm is not only suitable for CE precoding, but can also be applied to one-bit DACs precoding. Nevertheless, this approach must be used to approximate the SER expression of the system when formulating the optimization objective. Wang et al. [15] rotated and scaled each constellation point individually to take advantage of the additional degrees of freedom by jointly optimizing the transmit signal matrix and complex scaling factors to maximize the signal-to-interference-to-noise ratio (SINR) at the receiver.

Recently, researchers have found that including the concept of constructive interference (CI) in the design of precoding can significantly enhance system performance. Inspired by this, the authors in [16] investigated the utilization of CI to take advantage of the MUI in the system, with the aim of reducing the SER. In [17], a CEO-based method was proposed for PSK modulations, which achieved significant performance improvements over the classic CE precoding method based on interference minimization in [9]. Furthermore, Liu et al. [18] proposed an effective Riemannian conjugate gradient (RCG) method to address the CE precoding problem that takes into account CI and achieves a balance between performance and complexity. However, in fact, this CI-based CE precoding design is only suitable for PSK modulations. The CE characteristic of the signal assumes that the phase of the phase shifter is continuous or has nearly continuous phase resolution. Often, this is unsatisfactory. Therefore, it is necessary to study the discrete constant envelope (DCE) precoding methods in which the phase shifters have finite phase resolution. The investigation in [19] focused on the transmit signals in the DCE case. In [19], a PG-based symbol-level mean square error (MSE) precoding algorithm was proposed, which is not only applicable to the strict CE constraint, but also to the polygon constraint of DCE. In [20], a greedy precoding design using the MSE of system as the design criterion was proposed. The DCE precoder was solved in [21] when using a single common PA and separate digital phase shifters. This method combines DCE with CI to improve performance. Moreover, the authors in [11,14] have expanded their investigations to accommodate the DCE transmit signals.

As previously stated, CE precoding methods typically exhibit favorable performance. Motivated by this, our study focuses on the development of a CE precoding design that aims to minimize the difference between the received signal and transmit symbol in massive MU-MIMO systems. In comparison with [14], which focuses on designing the minimum SER of the system under the worst-case for a single user, our method aims to optimize the overall performance of the system. We address this by formulating a CE precoding problem using the minimum mean square error (MMSE) criterion. It is important to note that in [14], it is required to make an approximation of the objective problem, and we intentionally avoid doing so. The main contributions are as follows:

1. One of the main challenges in solving the CE precoding problem is the interdependence between the CE precoded signal and the precoding factor. To address this problem, we employ a two-stage iterative procedure involving an alternating minimization (AltMin) framework. When addressing the CE precoded signal, the CE constraint is simplified and transformed into unit modulus constraints by introducing an auxiliary variable. Additionally, the unit modulus constraint is converted to continuous by adding a penalty term to the objective function.
2. The optimal precoded signal is obtained using the majorization-minimization (MM) framework. In the MM framework, the key is how to construct the surrogate function. We exploit the channel characteristics of massive MU-MIMO systems and combine them with a second-order Taylor expansion to obtain an efficient surrogate function. Unlike the one-step GEMM method described in [14], we obtain the precise values of the auxiliary variables through multiple iterations. In addition, we derive the *L*-Lipschiz constant and analyze the exact property, convergence, and computational complexity of the proposed algorithm.
3. The proposed method is extended to DCE precoding schemes that have finite phase resolution. At first, we manipulate the continuous phase of the CE signal to align with the PSK constellation by performing a straightforward rotation. Then, we employ algebraic knowledge to derive the DCE precoded signal by making secondary decisions.
4. Simulation results demonstrate that in the CE precoding case, the proposed algorithm exhibits superior uncoded BER performance and a lower computational complexity when compared to existing approaches. In both PSK modulation and QAM modulation, the suggested CE precoding method can achieve a performance gain of about 1dB. In the 3-phase case, the proposed algorithm also has better performance.

The remainder of this paper is organized as follows: In Section 2, we present the model of the CE precoding system for the massive MU-MIMO system and the CE precoding problem based on the MMSE criterion. In Section 3, we give the detailed process of the algorithm for solving the CE precoding problem. Furthermore, the proposed algorithm is extended to the DCE case. The performance of the proposed algorithm is illustrated by analyzing its exact property, convergence, and computational complexity in Section 4. Section 5 presents the simulation, numerical results, and analysis. Section 6 is a summary of this paper.

*Notations:* In this paper, *a*, **a**, and A are the scalars, vectors, and matrices. For matrices and vectors, ${∥\cdot ∥}_{2}$ stands for the spectral norm and Euclidean norm, respectively. Operator $\left|\cdot \right|$ represents the absolute value of a scalar or the cardinality of a set. The transpose and its conjugate transpose of a vector or matrix are denoted by ${\left(\cdot \right)}^{T}$ and ${\left(\cdot \right)}^{H}$. $\langle a,b\rangle$ is the Euclidean inner product. $ℜ\left(\cdot \right)$ and $ℑ\left(\cdot \right)$ denote the real and imaginary parts of the vector or matrix. The $\left\lceil \cdot \right\rceil$ means rounding up to an integer. The set of complex numbers is denoted by C. The matrix I is an identity matrix with the appropriate dimensions.

## 2. System Model and Problem Formulation

### 2.1. System Model

**Figure 1:**
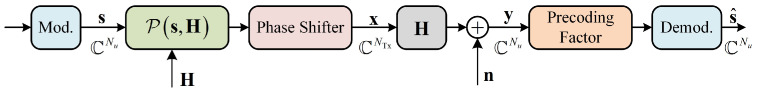
The CE precoder for the massive MU-MIMO downlink system.

As shown in Figure 1, we consider a single-cell, single-carrier massive MU-MIMO downlink transmission system in TDD mode, in which the BS is equipped with a large-scale antenna array $N_{\mathrm{TX}}$ and communicates with $N_{u}$ single-antenna users at the same time, that is $N_{\mathrm{TX}}\;\gg \;N_{u}$. At the BS, each RF chain is connected to a phase shifter, and it is assumed that the phase shifter can produce continuous phase values throughout the entire phase range. Therefore, the input constellation symbol vector $s={\left[s_{1},\ldots ,s_{N_{u}}\right]}^{T}\in O$ passes through the precoder and phase shifters to form a transmit signal $x={\left[x_{1},\ldots ,x_{N_{\mathrm{TX}}}\right]}^{T}\in C^{N_{\mathrm{TX}}}$ with a constant envelope, where O is the set of constellation points. The CE precoded signal transmitted by the *t*-th antenna at the BS is expressed as

(1) $$x_{t}=\sqrt{\frac{P_{T}}{N_{\mathrm{TX}}}}e^{j\theta_{t}},\forall t\in \left\{1,2,\ldots ,N_{\mathrm{TX}}\right\},$$

where $\theta_{t}\in \left[0,2\pi \right]$ is the phase of the CE precoded signal $x_{t}$. $P_{T}$ is the instantaneous transmit power, and this shows that the CE precoded signal satisfies ${∥x∥}_{2}^{2}\leq P_{T}$. The CE property forces the transmit signal x to satisfy the CE constraint, that is

(2) $$V_{1}=\left\{x\in C^{N_{\mathrm{TX}}}\left|\left|x_{t}\right|=\sqrt{\frac{P_{T}}{N_{t}}},t=1,\ldots ,N_{\mathrm{TX}}\right.\right\}.$$

Assuming a transmission time duration not exceeding the channel coherence time. For such a system, the discrete-time complex baseband signal received at users during the downlink of an arbitrary coherence interval can be expressed as

(3) y=Hx+n,

where $y\;=\;{\left[y_{1},\ldots ,y_{N_{u}}\right]}^{T}\;\in \;C^{N_{u}}$ denotes the received signal vector of all users. The matrix $H\in C^{N_{u}\times N_{\mathrm{TX}}}$ denotes the downlink channel, which is perfectly known at the BS. The vector $n\in C^{N_{u}}$ is an additive noise and $n∼CN\left(0,\sigma^{2}I\right)$.

### 2.2. Problem Formulation

In the CE precoding design, the task is to design the transmit signal x under the CE constraint so that the MSE between the transmit symbol s and its estimated value $\hat{s}$ is minimized. In order to facilitate the use of channel gain, an additional precoding factor ψ∈R [22] is introduced in the CE precoding design. The users can use the precoding factor ψ to obtain an estimate of the transmit symbol s from the received signal y, i.e., $\hat{s}=\psi y$. At the receiving end, the users can estimate the precoding factor for the block-fading channel using either pilot-based estimation or blind estimation [23], where a direct way to obtain the estimated precoding factor for the user is to use the pilots known at the user side. The MSE between the transmit symbol and its estimated value $\hat{s}$ can be obtained by

(4) $$E_{s}\left[{∥s-\hat{s}∥}_{2}^{2}\right]={∥s-\psi \mathrm{Hx}∥}_{2}^{2}+\psi^{2}N_{u}\sigma^{2},$$

where we restrict the precoder results in the same precoding factor ψ for all the users [24]. With this assumption, the MSE after precoding will be roughly the same for all users, which guarantees a certain degree of fairness among the users [22]. Thus, the CE precoding problem based on the MMSE criterion is stated as,

(5) $$\begin{array}{c} \min_{x,\psi }\;{∥s-\psi \mathrm{Hx}∥}_{2}^{2}+\psi^{2}N_{u}\sigma^{2}\\ \;\;s.t.\;x\in V_{1},\;\psi \in R. \end{array}$$

Since the CE constraint of the CE precoded signal is non-convex, the optimization problem (5) is non-convex. Generally, the optimization problem (5) is NP-hard. In addition, the mutually coupled CE precoded signal and precoding factor also bring difficulties for directly optimizing the CE precoding problem (5). Next, the CE precoded signal and precoding factor will be solved separately in an AltMin method, and an effective algorithm based on the MM framework will be proposed to obtain the CE precoded signal.

## 3. Majorization-Minimization Method for Constant Envelope Precoding

In order to effectively solve the coupling between the CE precoded signal x and precoding factors ψ, the AltMin framework is used to decouple the optimization problem (5) into two subproblems, in which ψ or x are solved alternately while maintaining correspondingly another variable x or ψ which is fixed. Specifically, each iterative process of applying the AltMin framework to solving the optimization problem (5) is expressed as the following two steps:

(6a) $$\begin{array}{c} \psi^{i+1}=\underset{\psi \in R}{\arg\min}\;{∥s-\psi Hx^{i}∥}_{2}^{2}+\psi^{2}N_{u}\sigma^{2}, \end{array}$$

(6b) $$\begin{array}{c} x^{i+1}=\underset{x\in V_{1}}{\arg\min}\;{∥s-\psi^{i+1}\mathrm{Hx}∥}_{2}^{2}+{\left(\psi^{i+1}\right)}^{2}N_{u}\sigma^{2}, \end{array}$$

where the algorithm alternately solves the precoding factor and the CE precoded signal until the stopping condition $\left|\mathrm{MS}E^{i+1}-\mathrm{MS}E^{i}\right|/\mathrm{MS}E^{i+1}\;\leq \epsilon_{O}$ is met.

In the first step of AltMin, that is to solve subproblem (6a), given the CE precoded signal x, expand and rewrite subproblem (6a) as a quadratic function about the precoding factor ψ

(7) $$\begin{array}{c} \;\;\;{∥s-\psi \mathrm{Hx}∥}_{2}^{2}+\psi^{2}N_{u}\sigma^{2}\\ =s^{H}s-2\psi s^{H}\mathrm{Hx}+\psi^{2}{∥\mathrm{Hx}∥}_{2}^{2}+\psi^{2}N_{u}\sigma^{2}. \end{array}$$

Setting the partial derivative of (7) with respect to the precoding factor ψ to 0, we can obtain

(8) $$\psi =\frac{ℜ\left\{s^{H}\mathrm{Hx}\right\}}{{∥\mathrm{Hx}∥}_{2}^{2}+N_{u}\sigma^{2}}.$$

Next, the CE precoded signal is optimized by solving subproblem (6b), taking into account the precoding factor ψ. When the precoding factor ψ is provided as a constant, $\psi^{2}N_{u}\sigma^{2}$ will be eliminated from Equation (6b). To simplify further processing, define the auxiliary variable u and make $x=\sqrt{P_{T}/N_{\mathrm{TX}}\;}u$. Let $\tilde{H}=\sqrt{P_{T}/N_{\mathrm{TX}}\;}\psi H$. Hence, the subproblem (6b) can be reformulated as

(9) $$\begin{array}{cc} \; & \min_{u}\;{∥s-\tilde{H}u∥}_{2}^{2}\\ \; & \;\;s.t.\;u\in V_{2}, \end{array}$$

where $V_{2}=\left\{u\in C^{N_{\mathrm{TX}}}\left|\left|u_{t}\right|=1,t=1,\ldots ,N_{\mathrm{TX}}\right.\right\}$. This means that any $u_{t}$ in $V_{2}$ is on the unit circle.

It can be seen from (9) that although the objective function to be optimized is quadratic, the constraint $V_{2}$ is a non-convex unit modulus constraint, so the problem is still non-convex. The generally employed approach for solving problems with a quadratic objective function is the semi-definite relaxation (SDR) method [25,26,27]. SDR has the advantage of employing a non-convex optimization problem to approximate the objective function. Although SDR is capable of calculating approximate solutions to non-convex optimization problems in polynomial time, the worst-case computational complexity is proportional to $N_{\mathrm{Tx}}^{4.5}$ [27], hindering its application to large-scale applications. To improve the effectiveness of solving the optimization problem (9), we can explore the implementation of the penalty method in the unit modulus optimization problem. This involves relaxing the unit modulus constraint set for solving the CE precoded signal x and incorporating a penalty function into the objective function to ensure that the solution lies on the unit circle [28].

Let $f\left(u\right)={∥s-\tilde{H}u∥}_{2}^{2}$, the optimization problem can be written as

(10) $$\min_{u\in V_{3}}\;F_{\rho }\left(u\right)=f\left(u\right)-\rho {∥u∥}_{2}^{2},$$

where $V_{3}=\left\{u\in C^{N_{\mathrm{TX}}}\left|\left|u_{t}\right|\leq 1,t=1,\ldots ,N_{\mathrm{TX}}\right.\right\}$. The ρ>0 is a penalty parameter. Since CE precoded signals are complex, the penalty term $-\rho {∥u∥}_{2}^{2}$ is used to push each $u_{t}$ to any position on the unit circle. It is important to note that while the constraint of the optimization problem (10) exhibits convexity, the objective function is non-convex. Hence, the optimization problem (10) may be classified as a convex constrained minimization problem with a non-convex objective function. This problem can be effectively tackled by employing first-order optimization techniques like the PG method. In general, first-order optimization approaches exhibit a modest level of iterative complexity; however, the iterations needed to attain convergence can be significant. In the theory of convex optimization, it is usual to employ Nesterov- or FISTA-type acceleration algorithms [29] as a way to minimize the number of iterations required. In addition, in large-scale MU-MIMO systems, the optimization problem (10) is usually a large-scale problem, which poses a challenge to using classic algorithms to solve this optimization problem, and the MM architecture can solve this problem [30]. Next, a novel algorithm derived from the MM framework and FISTA method is introduced to efficiently address the optimization problem (10).

### 3.1. Surrogate Function Using Second-Order Taylor Expansion

Before providing the proposed method, an overview of the fundamental concepts underlying the MM framework for addressing minimization problems is provided. The MM framework is used to iteratively solve a series of simpler problems to replace non-convex optimization problems that are difficult to solve directly [31,32,33]. For example, consider minimizing the function $J\left(w\right)$ within the feasible set w∈W. Minimizing the function $J\left(w\right)$ becomes challenging when the objective function or constraint are non-convex. Thus, rather than directly minimizing the function $J\left(w\right)$, the surrogate function $J\left(w\left|w^{k}\right.\right)$ of the original objective function is minimized during the *k*-th iteration. A valid surrogate function has the following properties:

(11a) $$\begin{array}{cc} \;\;\;\;\;\;\;\;\;\;J\left(w\left|w^{k}\right.\right) & >J\left(w\right),\forall w\in W, \end{array}$$

(11b) $$\begin{array}{cc} \;J\left(w^{k}\left|w^{k}\right.\right) & =J\left(w^{k}\right), \end{array}$$

(11c) $$\begin{array}{cc} \nabla J\left(w^{k}\left|w^{k}\right.\right) & =\nabla J\left(w^{k}\right). \end{array}$$

The above properties indicate that the surrogate function is a tight upper bound of the original objective function. Therefore, the algorithm based on the MM framework starts from the feasible initial point $w^{0}\in W$ and iteratively minimizes the surrogate function

(12) $$w^{k+1}=\underset{w\in W}{\arg\min}\;J\left(w\left|w^{k}\right.\right).$$

To effectively utilize the MM framework for solving problem (10), the crucial aspect is to construct the surrogate function for the objective function. The following Lemma 1 serves as the foundation for the efficient development of surrogate functions [34]. The detailed proofs are described in [35].

**Lemma** **1.** *Consider a quadratic function of the form $a^{H}\mathrm{Sa}$, where S is a positive semi-definite matrix, then the surrogate function of the $a^{H}\mathrm{Sa}$ function at point $a^{k}$ is $a^{H}\mathrm{Ta}+2ℜ\left(\langle a,\left(S-T\right)a^{k}\rangle \right)+\langle a^{k},\left(T-S\right)a^{k}\rangle$, where T is a positive semi-definite matrix and T≥S.*

For any quadratic differentiable function with bounded curvature, Lemma 1 is also known as the quadratic upper bound principle [32]. We first construct the surrogate function of $F_{\rho }\left(u\right)$. By expanding $F_{\rho }\left(u\right)$, we can obtain

(13) $$F_{\rho }\left(u\right)=s^{H}s-s^{H}\tilde{H}u-u^{H}{\tilde{H}}^{H}s+u^{H}{\tilde{H}}^{H}\tilde{H}u-\rho {∥u∥}_{2}^{2}.$$

According to Lemma 1, we consider alternatives $u^{H}{\tilde{H}}^{H}\tilde{H}u$ in (13). We define $S={\tilde{H}}^{H}\tilde{H}$, and consider the second-order Taylor expansion of $u^{H}\mathrm{Su}$ around $u^{k}$ as

(14) $$\begin{array}{c} u^{H}\mathrm{Su}=\langle u^{k},Su^{k}\rangle +\langle u^{k},S\left(u-u^{k}\right)\rangle +{\left(u-u^{k}\right)}^{H}Su^{k}+{\left(u-u^{k}\right)}^{H}S\left(u-u^{k}\right). \end{array}$$

For the massive MIMO system, the channel matrix H is a fat matrix, and S is a positive semi-definite Hermitian matrix. Based on the Lemma 1, we replace the matrix S with a matrix T, where T≥S. This implies that (14) is rewritten as

(15) $$\begin{array}{cc} u^{H}\mathrm{Su} & \leq \langle u^{k},Su^{k}\rangle +\langle u^{k},S\left(u-u^{k}\right)\rangle +{\left(u-u^{k}\right)}^{H}Su^{k}+{\left(u-u^{k}\right)}^{H}S\left(u-u^{k}\right)\\ \; & =u^{H}\mathrm{Tu}+u^{H}\left(S-T\right)u^{k}+\langle u^{k},\left(S-T\right)u\rangle +\langle u^{k},\left(T-S\right)u^{k}\rangle . \end{array}$$

Since S is a positive semi-definite Hermitian matrix, we perform eigenvalue decomposition on S and extract the maximum eigenvalue $\lambda_{\max}$, that is $\lambda_{\max}=\mathrm{eig}\left(S\right)$. We choose $T=\lambda_{\max}I$, then T≥S can be satisfied. Putting $T=\lambda_{\max}I$ into (15), we can obtain

(16) $$\begin{array}{c} u^{H}\mathrm{Su}\leq \lambda_{\max}u^{H}u+u^{H}\left(S-\lambda_{\max}I\right)u^{k}+\langle u^{k},\left(S-\lambda_{\max}I\right)u\rangle +\langle u^{k},\left(\lambda_{\max}I-S\right)u^{k}\rangle . \end{array}$$

We define

(17) $$\begin{array}{c} g\left(u\left|u^{k}\right.\right)=\lambda_{\max}u^{H}u+u^{H}\left(S-\lambda_{\max}I\right)u^{k}+\langle u^{k},\left(S-\lambda_{\max}I\right)u\rangle +\langle u^{k},\left(\lambda_{\max}I-S\right)u^{k}\rangle . \end{array}$$

Substituting $g\left(u\left|u^{k}\right.\right)$ into (13), we obtain the surrogate function $G\left(u{\left|u\right.}^{k}\right)$ of $F_{\rho }\left(u\right)$

(18) $$G_{\rho }\left(u{\left|u\right.}^{k}\right)=s^{H}s-s^{H}\tilde{H}u-u^{H}{\tilde{H}}^{H}s+g\left(u{\left|u\right.}^{k}\right)-\rho {∥u∥}_{2}^{2}.$$

It should be noted that, unlike the classical MM framework that approximates the non-convex part of the objective function, we use $T=\lambda_{\max}I$ to process $u^{H}{\tilde{H}}^{H}\tilde{H}u$ in (13) according to Lemma 1, and the resulting surrogate function $G_{\rho }\left(u{\left|u\right.}^{k}\right)$ is an upper bound on $F_{\rho }\left(u\right)$, which is a worst-case. The surrogate function $G_{\rho }\left(u{\left|u\right.}^{k}\right)$ satisfies the properties (Section 3.1). Next, we will use the above surrogate function and combine MM framework with the FISTA algorithm to iteratively solve the optimization problem (10).

### 3.2. MM Method for Solving CE Precoding

We take the general form of the MM method as shown below to find the minimum of the surrogate function $G_{\rho }\left(u{\left|u\right.}^{k}\right)$

(19) $$u^{k+1}=\underset{u\in V_{3}}{\arg\min}\;G_{\rho }\left(u\left|u^{k}\right.\right),k=0,1,2,\ldots$$

In the process of solving (19), we use the FISTA method [36] to solve. The FISTA method for solving $\min_{u\in V_{3}}\;G_{\rho }\left(u\left|u^{k}\right.\right)$ is

(20) $$u^{k+1}=\underset{V_{3}}{\Pi }\;\left(z^{k}-\mu^{-1}\nabla G_{\rho }\left(z^{k}{\left|u\right.}^{k}\right)\right),k=0,1,2,\ldots ,$$

where μ is the step size. Notably, $z^{k}-\mu^{-1}\nabla G_{\rho }\left(z^{k}\left|u^{k}\right.\right)$ does not always satisfy the CE constraint. Consequently, we project $z^{k}-\mu^{-1}\nabla G_{\rho }\left(z^{k}\left|u^{k}\right.\right)$ to the CE constraint set $V_{3}$, i.e.,

(21) $$\underset{V_{3}}{\Pi }\;\left(z^{k}-\mu^{-1}\nabla G_{\rho }\left(z^{k}\left|u^{k}\right.\right)\right)=e^{j∡\left(z^{k}-\mu^{-1}\nabla G_{\rho }\left(z^{k}\left|u^{k}\right.\right)\right)},$$

where *j* is the imaginary unit. The *∡* is the corresponding phase. The gradient vector of $G_{\rho }\left(z^{k}\left|u^{k}\right.\right)$ is

(22) $$\nabla G_{\rho }\left(z^{k}{\left|u\right.}^{k}\right)\;=\;2\left(\left(\lambda_{\max}\;-\;\rho \right)z^{k}\;+\;\left(S\;-\;\lambda_{\max}I\right)u^{k}\;-\;{\tilde{H}}^{H}s\right).$$

The $z^{k}$ is an extrapolated point and is updated with respect to $u^{k-1}$ from the previous iteration and $u^{k}$ from the current iteration

(23) $$z^{k}=u^{k}+\alpha_{k}\left(u^{k}-u^{k-1}\right),$$

with

(24) $$\alpha_{k}=\frac{\xi_{k-1}-1}{\xi_{k}},\;\;\xi_{k}=\frac{1+\sqrt{1+4\xi_{k-1}^{2}}}{2},$$

and with $\xi_{-1}=0$, $u^{-1}=u^{0}$. The ${\left\{\alpha_{k}\right\}}_{k\geq 0}$ is the extrapolation sequence. In particular, when $\alpha_{k}=0$ in (23), the FISTA method is simplified to the PG method.

For the step size, we choose to use the *L*-Lipschiz constant as the step size μ. When $f\left(u\right)$ is a convex function on ${\left[-1,1\right]}^{N_{\mathrm{TX}}}$, the Lipschitz continuity condition of $f\left(u\right)$ holds according to the following lemma:

**Lemma** **2.** *For a function $f\left(u\right)$ that is L-Lipschitz continuous in the domain ${\left[-1,1\right]}^{N_{\mathrm{TX}}}$, its L-Lipschitz constant is*

(25) $$L=2\sqrt{N_{\mathrm{TX}}}{∥\tilde{H}∥}_{2}^{2}.$$

**Proof.**  See Appendix A for a proof. □

In summary, the proposed MM (SoTMM) algorithm using the second-order Taylor expansion as the surrogate function uses (21) to (23) to iteratively minimize the upper bound of the objective function to solve the non-convex CE precoding problem (9) until the stopping condition ${∥u^{k+1}-u^{k}∥}_{2}^{2}\leq \epsilon_{I}$ is met. After obtaining the optimal value of u, the CE precoded signal can be obtained using the relationship $x=\sqrt{P_{T}/N_{\mathrm{TX}}\;}u$. As a convenience, Algorithm 1 provides a summary of the detailed procedures for resolving the optimization problem (5), which is divided into two iteration loops: the inner iteration loop used to solve the CE precoded signal x, with *k* as the index; and the outer iteration loop used to optimize the precoding factor ψ, with *i* as the index.
**Algorithm 1** SoTMM method for solving problem (5)**Input:** s, H, $\sigma^{2}$.1:**Initialization**: $x^{0}=0$;2:**Set**: i=0, $\epsilon_{O}>0$.3:**repeat**4:  Compute the precoding factor ψ by (8);5:  Let $\tilde{H}=\sqrt{P_{T}/N_{\mathrm{TX}}\;}\psi H$, and use (25) to calculate the Lipschitz constant;6:  **Set**: $u^{0}=u^{-1}=\left(1/\sqrt{P_{T}/N_{\mathrm{TX}}\;}\;\right)x^{i}$, $\xi_{-1}=0$, penalty parameter ρ>L, step size μ=L, k=0, $\epsilon_{I}>0$.7:  Define $S={\tilde{H}}^{H}\tilde{H}$ and extract the largest eigenvalue $\lambda_{\max}$ by eigenvalue decomposition of S;8:  **repeat**9:   Compute $\alpha_{k}$ and $\xi_{k}$ by (24);10:   Compute the extrapolated point $z^{k}$ by (23);11:   Compute the gradient vector $\nabla G_{\rho }\left(z^{k}\left|u^{k}\right.\right)$ by (22);12:   Compute the $z^{k}-\mu^{-1}\nabla G_{\rho }\left(z^{k}{\left|u\right.}^{k}\right)$ and update $u^{k+1}=e^{j∡\left(z^{k}-\mu^{-1}\nabla G_{\rho }\left(z^{k}\left|u^{k}\right.\right)\right)}$;13:   k←k+1;14:  **until** *A stopping criterion triggers.*15:  Reconstruction $x=\sqrt{P_{T}/N_{\mathrm{TX}}\;}u$;16:  i←i+1;17:**until** *A stopping criterion triggers.***Output:** x, ψ.

### 3.3. DCE Precoding

In previous studies, we assume that the phase shifters of the CE precoder can produce continuous phase values throughout the entire phase range, or that the phase shifters have approximately continuous phase resolutions. If the above two situations are not the case, we should consider the DCE precoding for phase shifters with a finite phase resolution. In what follows, we will extend the proposed SoTMM algorithm to DCE precoding design. In the DCE precoding case, the CE constraint $V_{1}$ of the transmit signal x will be discretized as

(26) $$X=\left\{e^{j\frac{2\pi }{2^{\kappa }}p}\left|p=1,2,\cdots 2^{\kappa }\right.\right\},$$

where κ is the discrete resolution of the phase shifter, that is, κ is a positive integer of κ≥2. Therefore, after using Algorithm 1 to obtain the optimal CE precoded signal x, each signal element in the CE precoded signal x needs to be discretized to the closest DCE constraint set X points.

**Figure 2:**
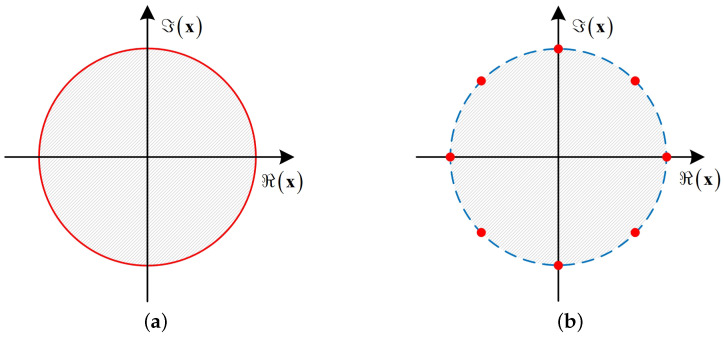
Illustration of constraint set. The red parts are the constraint set and the shaded parts are the relaxed constraint set. (**a**) CE set; (**b**) DCE set.

Figure 2 shows a diagram of the CE constraint set and the DCE constraint set, where the discrete resolution of the DCE constraint set is κ=3. The red parts represent the CE constraint set of CE precoding and the DCE constraint of DCE precoding, respectively, and the shaded parts represent the relaxed constraint set. As can be seen from Figure 2, the CE constraint can be regarded as a continuous point on a circle with a radius of $\sqrt{P_{T}/N_{\mathrm{TX}}\;}$, while the DCE constraint is a discrete point on the circle. Therefore, DCE precoding design is converted into a problem of how to design discrete phases on a circle, that is, designing a mapping method to discretize the continuous CE precoded signal into the DCE precoded signal. In general, it is difficult to obtain a strict algebraic expression of this mapping relationship. Existing research shows that in DCE precoding design, the CE precoded signal can be mapped to the $2^{\kappa }$-PSK constellation to obtain the DCE precoded signal [19].

**Figure 3:**
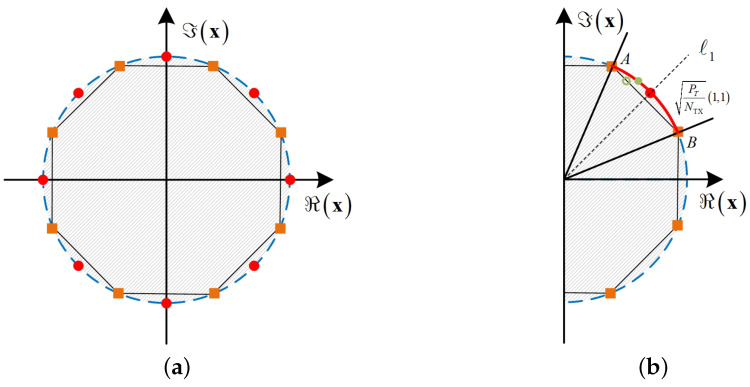
The projection of DCE precoding, κ=3. (**a**) DCE set, κ=3; (**b**) The projection onto the right half plane.

Using κ=3 as an example, Figure 3 illustrates the process of DCE precoding projection. The red points represent the DCE precoded signal, the green circles and points represent the projected DCE precoded signal, and the orange squares represent the 8-PSK constellation points. As can be seen from Figure 3a, the CE precoded signal obtained using Algorithm 1 satisfies the CE constraint, that is, the CE precoded signal all falls on the circle. When κ=3, the DCE precoded signal can be projected to the 8-PSK constellation point by simply rotating π/8 counterclockwise. According to Figure 3b, we will give a detailed projection process. During the projection process, a quadratic decision is used to make the mapping result more accurate. First, project the point located on $\overset{⌢}{AB}$ to the nearest point $\hat{x}$ on the straight line AB, that is

(27) $${\hat{x}}^{i+1}=\cos\left(\frac{2\pi }{2^{\kappa }}\right)+jℑ\left(x^{i+1}\right).$$

In order to determine the final projection point, the straight line equation is used to divide the straight line AB. Combining algebraic knowledge, the equation of the straight line $\ell_{1}$ can be obtained as

(28) $$\ell_{1}:ℑ\left(x\right)=ℜ\left(x\right).$$

Therefore, we can obtain the division of the straight line AB

(29) $$\mathrm{prox}\left({\hat{x}}^{i+1}\right)=\left\{\begin{array}{c} e^{j\frac{3\pi }{8}},ℑ\left({\hat{x}}^{i+1}\right)\geq \ell_{1},\\ e^{j\frac{\pi }{8}},ℑ\left({\hat{x}}^{i+1}\right)<\ell_{1}, \end{array}\right.$$

where $\mathrm{prox}\left(\cdot \right)$ means discretizing the input signal. Using the above formula, the CE precoded signal can be discretized into an 8-PSK constellation. Combining (27)–(29), the discrete expression of DCE precoding for arbitrary phases is given as

(30) $$\mathrm{prox}\left({\hat{x}}^{i+1}\right)=\left\{\begin{array}{c} e^{j\left(\frac{2\pi }{2^{\kappa }}+\frac{\left(n-1\right)\pi }{2^{\kappa }}\right)},ℑ\left({\hat{x}}^{i+1}\right)\geq \ell_{n-1},\\ e^{j\left(\frac{2\pi }{2^{\kappa }}-\frac{\left(n-1\right)\pi }{2^{\kappa }}\right)},ℑ\left({\hat{x}}^{i+1}\right)<\ell_{n-1}, \end{array}\right.$$

where $n=\left\lceil \frac{∡x+\pi /2^{\kappa }\;}{2\pi /2^{\kappa }\;}\right\rceil$. The point on the arc projects to the nearest point $\hat{x}$ on the corresponding straight line as

(31) $${\hat{x}}^{i+1}=\cos\left(\frac{n\pi }{2^{\kappa }}\right)+jℑ\left(x^{i+1}\right).$$

The straight line $\ell_{n-1}$ is given by

(32) $$ℑ\left(x\right)=\cos\left(\frac{n\pi }{2^{\kappa }}\right)ℜ\left(x\right).$$

To summarize, by utilizing (30)–(32), it is possible to obtain the DCE precoded signal with arbitrary phases. Finally, the DCE precoding factor is recalculated according to (8).

## 4. Performance Analysis

In this section, the exact property of the penalty optimization problem (10), the convergence performance, and the computational complexity of the proposed SoTMM algorithm are analyzed in detail.

### 4.1. The Exact Property of Problem (10)

For the minimization problem (10), it is natural to question whether the penalty optimization problem is an exact restatement of the original optimization problem (9). The following Theorem 1 [14] can illustrate this problem.

**Theorem** **1.** *Assume that the function f is Lipschitz continuous in the feasible set $V_{3}$. Then, there is a constant $\bar{\rho }>0$ such that for any $\rho >\bar{\rho }$, any (global) optimal solution to the optimization problem (10) is also the (global) optimal solution to the optimization problem (9). Especially, in the CE precoding case, $\bar{\rho }=L$, where L is the Lipschitz constant of function f in $V_{3}$.*

Theorem 1 shows that when the penalty parameter is large enough, the optimal solutions of problems (10) and (9) are equivalent. In particular, this equivalent result does not require additional dynamic adjustment of the penalty parameter ρ, which also provides a theoretical basis for the selection of the penalty parameter ρ.

### 4.2. Convergence Analysis

In fact, the convergence analysis of non-convex first-order methods involving the accelerated proximal gradient method or the FISTA method is challenging. Here, we are inspired by [14] to prove the convergence performance of the SoTMM algorithm using gradient extrapolation. Theorem 2 describes the convergence performance of the proposed SoTMM algorithm.

**Theorem** **2.** *Suppose there is a Lipschitz constant $L_{F}$ such that the function F has a Lipschitz continuous gradient. And for any $\bar{u}\in V_{3}$, there is a Lipschitz constant $L_{G}$ such that the surrogate function $G_{\rho }\left(\cdot \left|\bar{u}\right.\right)$ has a Lipschitz continuous gradient. In addition, assuming that $\alpha_{k}$ obtained from the (21) to (23) satisfies $0\leq \alpha_{k}\leq \bar{\alpha }$, making*

(33) $$\begin{array}{cc} \; & \min_{m=0,\cdots ,k}\;\mathrm{dist}\left(0,\nabla F\left(u^{m+1}\right)+\partial I_{V_{3}}\left(u^{m+1}\right)\right)\\ \; & \;\;\;\;\;\;\;\leq C\sqrt{\frac{8}{\left(k+1\right)\left(1-{\bar{\alpha }}^{2}\right)\mu }\left(F\left(u^{0}\right)-F^{*}\right)} \end{array}$$

*is true, then the proposed SoTMM is guaranteed to find a stationary point, where*

(34) $$C=\max\left(\left(L_{G}+\mu \right)\bar{\alpha },L_{F}+\mu \right).$$

**Proof.**  See Appendix B for a proof. □

### 4.3. Complexity Analysis

To illustrate the computational complexity of the SoTMM algorithm, we discuss the number of multiplications performed by the algorithm. First of all, it needs to be made clear that the SoTMM algorithm requires two iteration loops, inner and outer. The main complexity of the inner iteration loop comes from computing the gradient vector $\nabla G_{\rho }\left(z^{k}\left|u^{k}\right.\right)$ of the surrogate function $G_{\rho }\left(z^{k}\left|u^{k}\right.\right)$. The gradient computation in Lines 11–12 of Algorithm 1 is $N_{\mathrm{TX}}^{2}+N_{\mathrm{TX}}N_{u}+3N_{\mathrm{TX}}$, where the computational complexity of the projection operation is ignored. In the inner iteration loop, the eigenvalue decomposition of matrix S and the calculation of the extrapolation point $z^{k}$ require $N_{\mathrm{TX}}^{2}$ and $N_{\mathrm{TX}}$ multiplications, respectively. In Algorithm 1, lines 5, 6, and 14 are the relevant steps of the outer iteration loop, requiring a total of $N_{\mathrm{TX}}N_{u}+2N_{\mathrm{TX}}$ multiplication operations. In addition, in Algorithm 1, calculating the precoding factor ψ requires $N_{\mathrm{TX}}^{2}+N_{\mathrm{TX}}N_{u}+N_{\mathrm{TX}}$ complex multiplications. Hence, the overall computational complexity required to execute the proposed SoTMM algorithm once to obtain the optimal CE precoded signal and precoding factor is equal to

(35) $$O\left(K_{1}\left(2N_{\mathrm{TX}}^{2}+2N_{\mathrm{TX}}N_{u}+3N_{\mathrm{TX}}+K_{2}\left(N_{\mathrm{TX}}^{2}+N_{\mathrm{TX}}N_{u}+4N_{\mathrm{TX}}\right)\right)\right),$$

where $K_{1}$ and $K_{2}$ are the maximum number of iterations for the inner and outer iteration loops, respectively.

## 5. Simulation Results and Discussions

**Figure 4:**
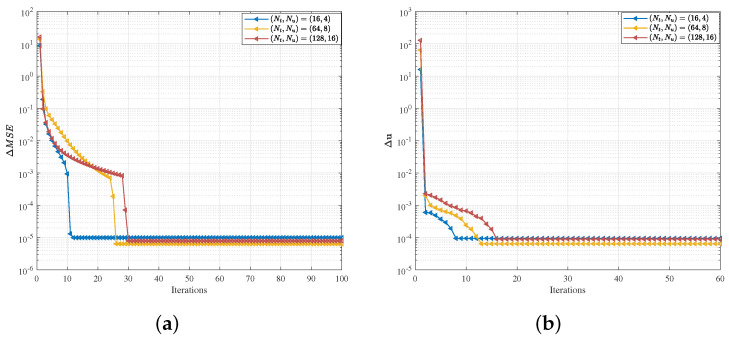
Convergence performance of the SoTMM algorithm with different system scales. (**a**) Outer iteration convergence performance; (**b**) Inner iteration convergence performance.

We conduct simulation experiments to validate the performance of the proposed SoTMM algorithm and compare it to several existing CE precoding schemes. Among the involved comparison algorithms are the ZF precoding scheme employing direct projection to the CE constraint (ZF-CE), the GP-AltMin [11], the FPG method [13], and the GEMM algorithm [14] (simulation evaluations are carried out utilizing the simulation parameters as suggested in [11,13,14]). Moreover, as a benchmark, we consider a ZF precoding scheme without CE constraint (ZF-non). The simulations consider the commonly used massive MU-MIMO downlink wireless communication system. The specific simulation conditions are set as follows: Assuming that the communication channel H between the BS and the users is a standard complex Gaussian channel, that is, $H∼CN\left(0,I\right)$. SNR is defined as $\mathrm{SNR}=P_{T}/\sigma^{2}$, where the transmit power is normalized to 1. All the simulation results are the average of $10^{3}$ Monte Carlo simulations. According to Theorem 1, the penalty parameter ρ>L and the step size μ=L are set in the proposed SoTMM algorithm. In particular, in Figure 4, one simulation experiment is enough to illustrate the convergence performance of the SoTMM algorithm.

### 5.1. Convergence Analysis

Figure 4 depicts the inner and outer convergence performance of the proposed SoTMM algorithm in different system settings when SNR=5dB and 16QAM modulation. The convergence performance of the SoTMM algorithm is explained by checking the iteration gap of the inner and outer iteration stop conditions, respectively, that is,

(36) $$\Delta \mathrm{MSE}=\frac{\mathrm{MS}E^{i+1}-\mathrm{MS}E^{i}}{\mathrm{MS}E^{i+1}},$$

(37) $$\Delta u={∥u^{k+1}-u^{k}∥}_{2}^{2}.$$

It can be concluded from Figure 4a that the SoTMM algorithm using the AltMin framework can achieve convergence in systems with different scales. As the number of BS antennas increases, the number of iterations required for the SoTMM algorithm to achieve convergence also increases. When the iterations exceed 30, the iteration gap of the SoTMM algorithm with different system sizes converge to $10^{-5}$. Figure 4b illustrates the convergence of the inner iteration loop using the MM framework to solve u. As can be seen from Figure 4b, similar to the outer iteration situation, as the number of BS antennas increases, more iterations are required for Δu to converge to $10^{-4}$. Nonetheless, it only takes about 20 iterations for Δu to converge to $10^{-4}$ across different system sizes. Therefore, in the following simulations, the maximum iteration of the outer iteration loop is set to 40, and the maximum iteration of the inner iteration loop is set to 20.

### 5.2. CE Precoding

**Figure 5:**
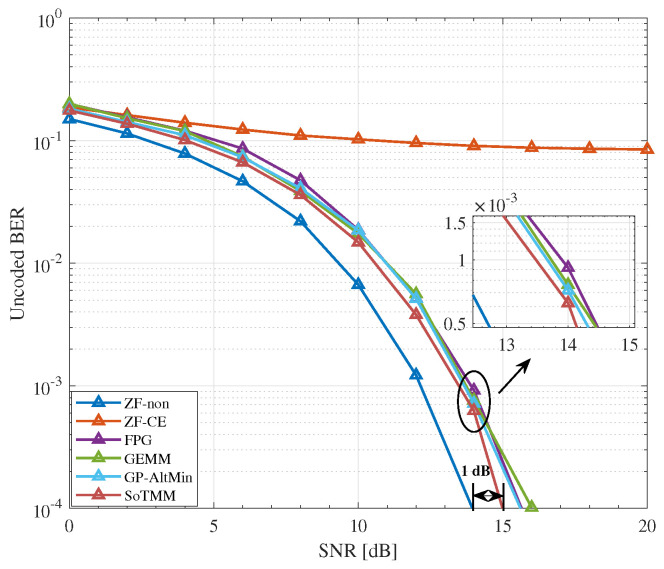
The uncoded BER performance for different CE precoders in $\left(N_{\mathrm{TX}},N_{u}\right)=\left(128,16\right)$ system, 16PSK.

**Figure 6:**
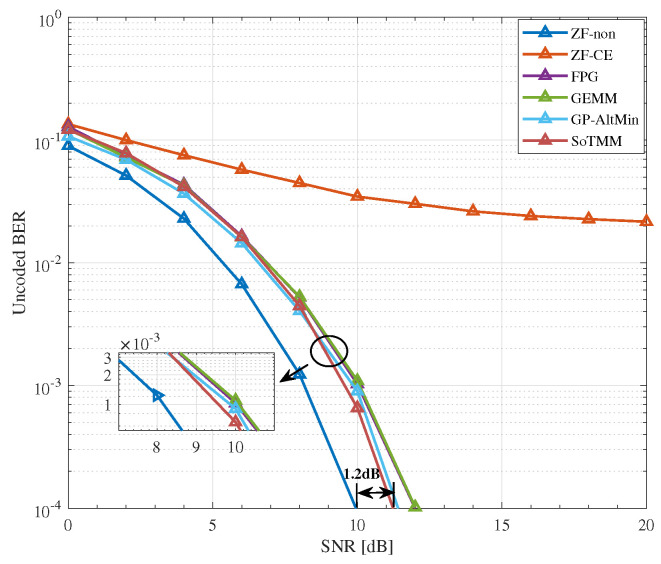
The uncoded BER performance for different CE precoders in $\left(N_{\mathrm{TX}},N_{u}\right)=\left(128,16\right)$ system, 16QAM.

**Figure 7:**
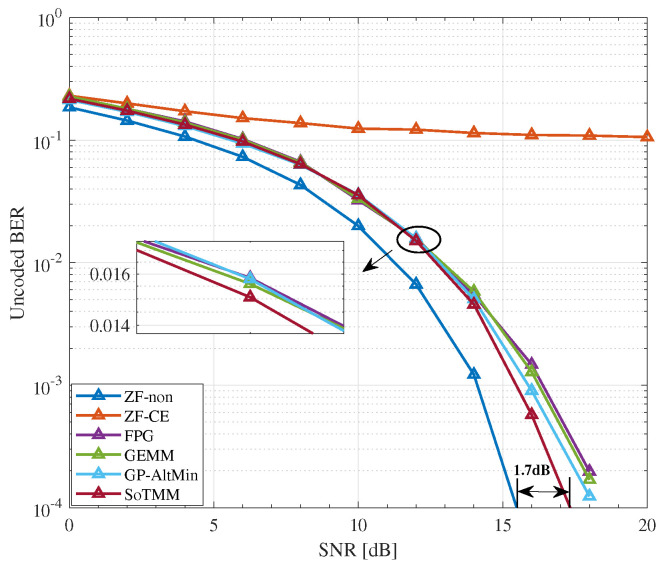
The uncoded BER performance for different CE precoders in $\left(N_{\mathrm{TX}},N_{u}\right)=\left(128,16\right)$ system, 64QAM.

We compare the uncoded BER performance of the proposed SoTMM algorithm in the massive MU-MIMO system, where the BS is equipped with 128 transmit antennas to communicate with 16 single antenna users. First, we compare the uncoded BER performance of the algorithm when the transmit symbol is generated by the constant modulus constellation, that is, 16PSK. It can be seen from Figure 5 that the performance of the proposed SoTMM algorithm is better than that of FPG, GEMM, and GP-AltMin, and the SNR gap between the SoTMM and the ideal ZF is only 1dB, which is promising. Different from PSK modulation, which can easily generate CE transmission signals, we pay more attention to the performance of the proposed algorithm in non-constant modulus modulation. Therefore, Figure 6 and Figure 7 verify the performance of the proposed algorithm when the input constellation symbols s are generated by 16QAM and 64QAM modulation, respectively. From Figure 6, several conclusions can be drawn. First of all, compared with the transmit symbols using 16PSK modulation, the performance of all algorithms in the non-constant modulus of 16QAM is improved, possibly because the distance between the constellation points of 16PSK is closer than that of 16QAM in the case of normalized transmit power, so the system performance will be reduced. Second, the ZF-CE precoding that directly projects the ZF precoded signal to the CE constraint has the worst performance, which is related to the limitations of the linear ZF precoding itself. Compared with the FPG and GEMM precoders, the proposed SoTMM precoder has the best performance. As can be seen from Figure 6, when the SNR exceeds 8dB, as the SNR further increases, the performance advantage of the SoTMM precoder gradually becomes prominent. Compared with the ideal ZF precoding, the performance gap between the two precoders is about 1.2dB. Finally, it can be seen from Figure 6 that the performance of the SoTMM and the GP-AltMin are similar. However, the GP-AltMin ignores the impact of noise when solving the CE precoded signals, thus improving performance. When considering the impact of noise on the CE precoded signals, the performance of the GP-AltMin needs to be further confirmed. When the constellation symbols s are generated using 64QAM modulation, the performance of all CE precoders decreases, among which the ZF-CE suffers an obvious performance loss. Similar to the 16QAM modulation situation, the proposed SoTMM precoder still has the lowest BER performance. When the uncoded BER is $10^{-4}$, the uncoded BER performance gap between the ideal ZF precoding and the SoTMM is only 1.7dB. This shows that the proposed SoTMM precoder has superior gains in uncoded BER performance compared with other CE precoders.

### 5.3. DCE Precoding

**Figure 8:**
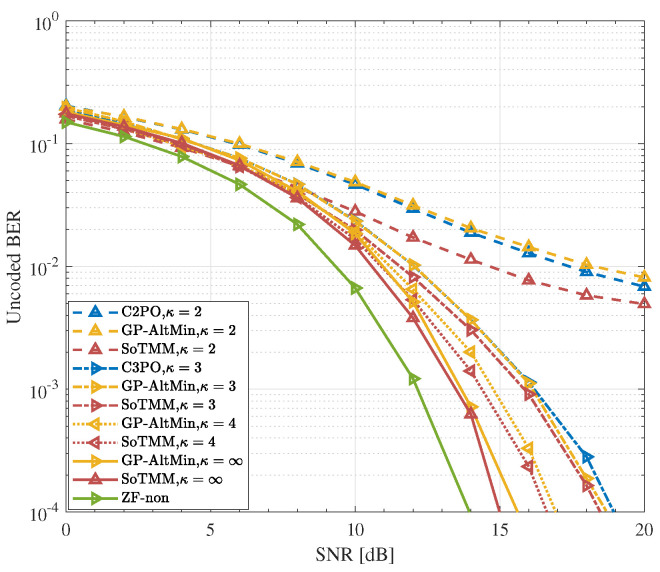
The uncoded BER performance for different CE precoders in different DCE cases. $\left(N_{\mathrm{TX}},N_{u}\right)=\left(128,16\right)$ system, 16PSK.

**Figure 9:**
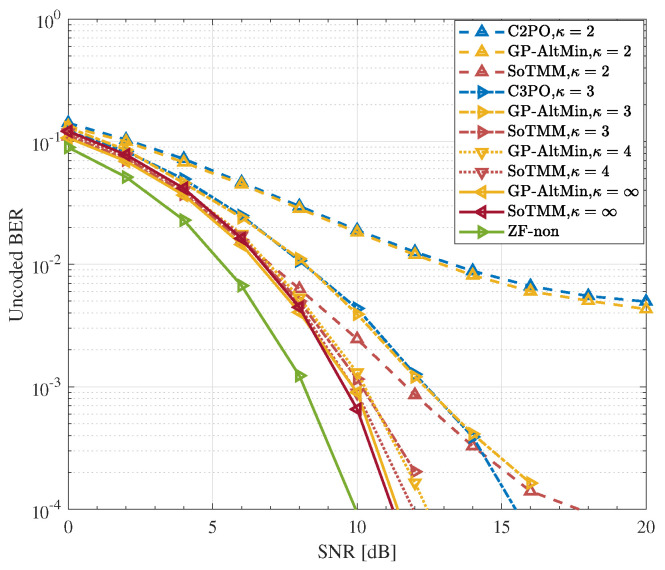
The uncoded BER performance for different CE precoders in different DCE cases. $\left(N_{\mathrm{TX}},N_{u}\right)=\left(128,16\right)$ system, 16QAM.

**Figure 10:**
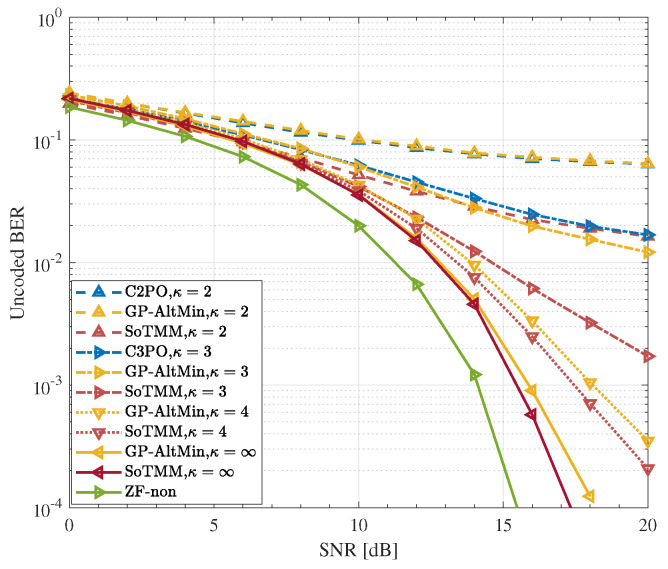
The uncoded BER performance for different CE precoders in different DCE cases. $\left(N_{\mathrm{TX}},N_{u}\right)=\left(128,16\right)$ system, 64QAM.

In the previous simulations, the performance of the proposed SoTMM algorithm under CE constraint was verified. Next, we apply the SoTMM algorithm to the DCE constraint case to further elaborate on the performance of the proposed SoTMM algorithm. Figure 8, Figure 9 and Figure 10 verify the uncoded BER performance of the SoTMM algorithm in different DCE cases. The simulations consider that the BS is equipped with 128 transmit antennas to communicate with 16 single antenna users. The constellation symbols s are generated by 16PSK, 16QAM, and 64QAM modulations, respectively. Some conclusions can be drawn from Figure 8, Figure 9 and Figure 10. First, it can be seen from Figure 8 that, similar to the CE precoding case, the uncoded BER of the algorithm is slightly higher when the input symbol s is generated by 16PSK compared with 16QAM. Secondly, as the discrete resolution κ of the phase shifter increases, the uncoded BER performance of all algorithms continues to improve. Secondly, when the discrete resolutions are κ=2 and κ=3, the uncoded BER performance of the C2PO (C3PO) and the GP-AltMin are almost the same, and are significantly worse than the SoTMM. In particular, in Figure 9, when the discrete resolution is κ=3, the uncoded BER performance of the proposed SoTMM is improved by about 3dB compared with the C3PO and GP-AltMin. Third, when the discrete resolution of the phase shifter is raised to κ=4, both the GP-AltMin and the SoTMM exhibit comparable uncoded BER performance, which closely approximates the performance achieved by the method when using a phase shifter with infinite resolution. Fourth, Figure 10 demonstrates that when the modulation is converted from 16QAM to 64QAM modulation, the uncoded BER performance of the 2-phase and 3-phase GP-AltMin and C2PO (C3PO) will change to unacceptable levels and gradually approach saturation. This error-floor problem can be greatly mitigated by increasing the discrete resolution. Even in the 2-phase and 3-phase cases of 64QAM modulation, the proposed SoTMM still has better uncoded BER performance. Finally, unlike the C2PO (C3PO) algorithm for specific discrete phases, the SoTMM algorithm is suitable for DCE precoding design with arbitrary phase shifter discrete resolution. In conclusion, the suggested SoTMM algorithm, as demonstrated in Figure 8, Figure 9 and Figure 10, is more suitable for practical applications compared to the existing CE precoding technique and can be more flexibly extended to DCE precoding design.

### 5.4. Complexity Analysis

**Figure 11:**
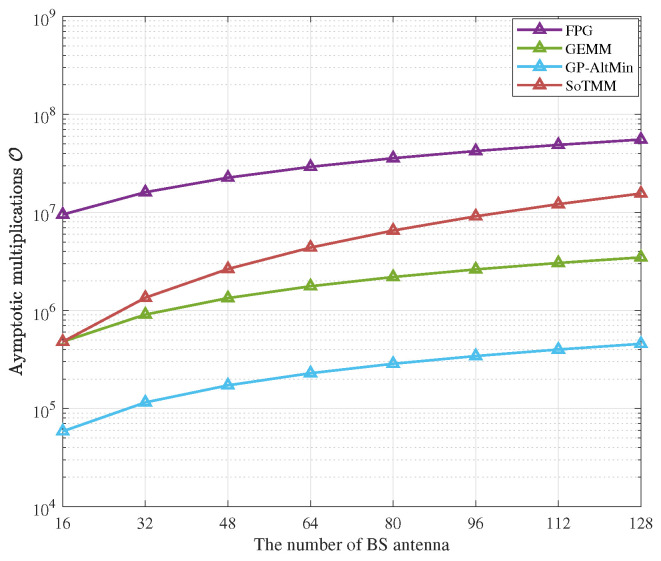
Comparison of computational complexity of different CE precoding algorithms, $N_{u}=16$.

In this subsection, we elucidate the complexity of the proposed SoTMM algorithm by quantifying the number of complex multiplications involved in the SoTMM precoding method within MU-MIMO systems of varying dimensions. The number of users in the massive MU-MIMO systems is set to 16. Table 1 displays the computational complexity of several CE precoding technologies. Figure 11 presents a comparison of the number of complex multiplications used by various CE precoding techniques in variable BS transmit antenna systems. Based on the findings presented in Figure 11, it is evident that the GP-AltMin exhibits the least computational complexity, while the GEMM follows closely behind. This is because only one-step projected gradient method is used in the GP-AltMin to optimize the CE precoded signal. However, the performance of algorithms using one-step projected gradient methods is usually unsatisfactory. The GP-AltMin artificially ignores the noise in the system to improve performance. However, improving the GP-AltMin in systems affected by noise may bring additional computational complexity. It is worth noting that while the GP-AltMin technique has low computational complexity, the SoTMM approach, as seen in Figure 9 and Figure 10, can be more efficiently used for DCE case design. Compared with the GEMM algorithm, although the proposed SoTMM algorithm requires more computational complexity in the $\left(N_{\mathrm{TX}},N_{u}\right)=\left(128,16\right)$ system. Nevertheless, when considering the prior examination of uncoded BER performance, it is evident that the SoTMM method outperforms the GEMM algorithm in terms of uncoded BER performance (as shown in Figure 7, the performance gap is about 1dB). Out of all the CE precoding techniques, FPG has the greatest computational complexity. This is because, as can be seen from Table 1, although the computational complexity of one iteration of the FPG algorithm is modest, in order to obtain the best performance, the FPG requires more iterations. This leads directly to the overall computational complexity of the FPG algorithm being too high. As a conclusion, the proposed SoTMM algorithm can efficiently strike a balance between uncoded BER performance and complexity.

## 6. Conclusions

In this paper, a novel CE precoding scheme for massive MU-MIMO downlink systems is proposed. Different from existing methods, the CE precoded signal and precoding factor are designed to make the received signal approximate the transmit symbol as much as possible. This algorithm employs an AltMin framework that combines the MM method and the FISTA method to iteratively optimize variables. In particular, the second-order Taylor expansion and the properties of the massive MU-MIMO channel are employed to formulate a surrogate function that is effective in facilitating implementation of the MM technique. Furthermore, this approach is expanded into the DCE precoding design. This work thoroughly examines the exact property, convergence, and computational complexity of the suggested algorithm. The simulation findings demonstrate that this algorithm exhibits favorable uncoded BER performance and possesses computational efficiency, whether in the CE precoding case or DCE precoding case. In future, we intend to expand the scope of our work to a wider range of applications, such as combining low-resolution digital-to-analog converters precoding and CE transmission to further reduce the power consumption of the system.

## Figures and Tables

**Table 1:** The complex multiplication of different CE precoding algorithms.

Methods	Maximum Iterations	Computational Complexity
FPG	K=5000	$O\left(K\left(5N_{\mathrm{TX}}N_{u}+2N_{u}^{2}+2N_{\mathrm{TX}}+5N_{u}\right)\right)$
GEMM	K=400	$O\left(K\left(N_{\mathrm{TX}}N_{u}+3N_{\mathrm{TX}}+8N_{u}\right)\right)$
GP-AltMin	$K_{1}=20$, $K_{2}=5$	$O\left(N_{\mathrm{TX}}N_{u}+K_{1}\left(N_{\mathrm{TX}}N_{u}+N_{\mathrm{TX}}+K_{2}\left(2N_{\mathrm{TX}}N_{u}+N_{u}\right)\right)\right)$
SoTMM	$K_{1}=40$, $K_{2}=20$	$O\left(K_{1}\left(2N_{\mathrm{TX}}^{2}+2N_{\mathrm{TX}}N_{u}+3N_{\mathrm{TX}}+K_{2}\left(N_{\mathrm{TX}}^{2}+N_{\mathrm{TX}}N_{u}+4N_{\mathrm{TX}}\right)\right)\right)$

## Data Availability

The data presented in this study are available on request from the corresponding author.

## Appendix A. Proof of Lemma 2

First, we can find that $f_{\rho }\left(u\right)$ is continuous in ${\left[-1,1\right]}^{N_{\mathrm{TX}}}$, so it is differentiable everywhere. Suppose $u_{1}$ and $u_{2}$ are two different input variables of $f_{\rho }\left(u\right)$, and satisfy $-1\leq u_{1}\leq 1$ and $-1\leq u_{2}\leq 1$, then, we can obtain

(A1) $$\left|f\left(u_{1}\right)-f\left(u_{2}\right)\right|=\left|{∥s-\tilde{H}u_{1}∥}_{2}^{2}-{∥s-\tilde{H}u_{2}∥}_{2}^{2}\right|.$$

Using the matrix/vector operator norm inequality, we can obtain the following inequality

(A2) $$\begin{array}{cc} \left|f\left(u_{1}\right)-f\left(u_{2}\right)\right| & =\left|{∥s-\tilde{H}u_{1}∥}_{2}^{2}-{∥s-\tilde{H}u_{2}∥}_{2}^{2}\right|\\ \; & \leq {∥\left(s-\tilde{H}u_{1}\right)-\left(s-\tilde{H}u_{2}\right)∥}_{2}^{2}\\ \; & \leq {∥\tilde{H}∥}_{2}^{2}{∥u_{1}-u_{2}∥}_{2}^{2}. \end{array}$$

On the other hand, since $-1\leq u_{1}\leq 1$ and $-1\leq u_{2}\leq 1$ hold, there is always

(A3) $${∥u_{1}-u_{2}∥}_{2}\leq 2\sqrt{N_{\mathrm{TX}}}.$$

The equality holds if, and only if, $u_{1}$ or $u_{2}$ equals 1 and the other variable equals −1. Substituting (A3) into the last inequality of (A2), we can obtain the upper bound of $\left|f\left(u_{1}\right)-f\left(u_{2}\right)\right|$ as

(A4) $$\left|f\left(u_{1}\right)-f\left(u_{2}\right)\right|\leq 2\sqrt{N_{t}}{∥\tilde{H}∥}_{2}^{2}{∥u_{1}-u_{2}∥}_{2}.$$

According to $\left|f_{\rho }\left(u_{1}\right)-f_{\rho }\left(u_{2}\right)\right|/{∥u_{1}-u_{2}∥}_{2}$ [37], we can obtain that (A4) is bounded as

(A5) $$L=2\sqrt{N_{\mathrm{TX}}}{∥\tilde{H}∥}_{2}^{2},$$

where *L* is a positive constant with ${∥\tilde{H}∥}_{2}^{2}\neq 0$.

## Appendix B. Proof of Theorem 2

It is pointed out in [38] that gradient iteration $u^{k+1}=z^{k}-\mu^{-1}\nabla_{u}G_{\rho }\left(z^{k}\left|u^{k}\right.\right)$ can be regarded as the proximal regularization of the linearized function G at $x^{k}$ [39]. Therefore, in the proposed SoTMM algorithm, the updated $u^{k+1}$ in (20) can be equivalently rewritten as

(A6) $$u^{k+1}=\min_{u}\;\frac{\mu_{k}}{2}{∥u^{k}-\left(z^{k}-\frac{1}{\mu }\nabla_{u}G_{\rho }\left(z^{k}\left|u^{k}\right.\right)\right)∥}_{2}^{2}+I_{V_{3}}\left(u\right),$$

where $I_{V_{3}}\left(\cdot \right)$ is the indicator function, that is, when $u\in V_{3}$, $I_{V_{3}}\left(u\right)=0$; when $u\notin V_{3}$, $I_{V_{3}}\left(u\right)=\infty$. The first-order optimality condition of (A6) at point $u^{k+1}$ is

(A7) $$\mu \left(u^{k+1}-z^{k}\right)+\nabla_{u}G_{\rho }\left(z^{k}\left|u^{k}\right.\right)+\partial I_{V_{3}}\left(u^{k+1}\right)=0.$$

We assume $v^{k+1}\in \partial I_{V_{3}}\left(u^{k+1}\right)$, and define $\nabla F\left(u^{k+1}\right)=\mu \left(u^{k+1}-z^{k}\right)+\nabla_{u}G_{\rho }\left(z^{k}\left|u^{k}\right.\right)$ according to (20), then, $v^{k+1}=-\nabla F\left(u^{k+1}\right)$. We can further rewrite (A7) as

(A8) $$\begin{array}{cc} \; & \;\;\;\;\;\;\mathrm{dist}\left(0,\mu \left(u^{k+1}-z^{k}\right)+\nabla_{u}G_{\rho }\left(z^{k}\left|u^{k}\right.\right)+\partial I_{V_{3}}\left(u^{k+1}\right)\right)\\ \; & \leq {∥\mu \left(u^{k+1}-z^{k}\right)+\nabla_{u}G_{\rho }\left(z^{k}\left|u^{k}\right.\right)+v^{k+1}∥}_{2}\\ \; & ={∥\mu \left(u^{k+1}-z^{k}\right)+\nabla_{u}G_{\rho }\left(z^{k}\left|u^{k}\right.\right)-\nabla F\left(u^{k+1}\right)∥}_{2}\\ \; & \leq \underset{\mathrm{first}\;\mathrm{term}}{\underset{︸}{{∥\nabla_{u}G_{\rho }\left(z^{k}\left|u^{k}\right.\right)-\nabla F\left(u^{k+1}\right)∥}_{2}}}+\underset{\mathrm{second}\;\mathrm{term}}{\underset{︸}{{∥\mu \left(u^{k+1}-z^{k}\right)∥}_{2}}}, \end{array}$$

where $\mathrm{dist}\left(a,\chi \right)=\inf_{b\in \chi }{∥a-b∥}_{2}$. Next, we will illustrate the convergence of the algorithm by analyzing the characteristics of the first and second terms of (A8). First, the first term of (A8) can be written as

(A9) $$\begin{array}{cc} \; & \;\;\;\;\;\;{∥\nabla_{u}G_{\rho }\left(z^{k}\left|u^{k}\right.\right)-\nabla F\left(u^{k+1}\right)∥}_{2}\\ \; & \;\overset{\left(a\right)}{=}\;{∥\nabla_{u}G_{\rho }\left(z^{k}\left|u^{k}\right.\right)-\nabla_{u}G_{\rho }\left(u^{k+1}\left|u^{k+1}\right.\right)∥}_{2}\\ \; & \;\leq {∥\nabla_{u}G_{\rho }\left(z^{k}\left|u^{k}\right.\right)-\nabla_{u}G_{\rho }\left(u^{k}\left|u^{k}\right.\right)∥}_{2}+{∥\nabla_{u}G_{\rho }\left(u^{k}\left|u^{k}\right.\right)-\nabla_{u}G_{\rho }\left(u^{k+1}\left|u^{k+1}\right.\right)∥}_{2}\\ \; & \;\overset{\left(b\right)}{\leq }\;L_{G}{∥z^{k}-u^{k}∥}_{2}+L_{F}{∥u^{k}-u^{k+1}∥}_{2}\\ \; & \;\overset{\left(c\right)}{=}\;L_{G}\alpha_{k}{∥u^{k}-u^{k-1}∥}_{2}+L_{F}{∥u^{k}-u^{k+1}∥}_{2}, \end{array}$$

where $L_{G}$ is the Lipschitz constant of $G_{\rho }$, and $L_{F}$ is the Lipschitz constant of F. In the derivation of (A9), (a) is derived from $\nabla_{u}G_{\rho }\left(u\left|u\right.\right)=\nabla F\left(u\right)$; (b) is derived from the Lipschitz continuity of $\nabla F\left(u\right)$ and $\nabla_{u}G_{\rho }\left(u\left|u\right.\right)$; (c) is derived from (23).

The second term in (A8) is analyzed below. According to (23), we can obtain

(A10) $$\begin{array}{cc} {∥\mu \left(u^{k+1}-z^{k}\right)∥}_{2} & =\mu {∥u^{k}-u^{k+1}+\alpha_{k}\left(u^{k}-u^{k-1}\right)∥}_{2}\\ \; & \leq \mu {∥u^{k}-u^{k+1}∥}_{2}+\mu \alpha_{k}{∥u^{k}-u^{k-1}∥}_{2}. \end{array}$$

Combining (A8)–(A10), we can obtain

(A11) $$\begin{array}{cc} \; & \;\;\;\;\;\mathrm{dist}\left(0,\mu \left(u^{k+1}-z^{k}\right)+\nabla_{u}G_{\rho }\left(z^{k}{\left|u\right.}^{k}\right)+\partial I_{V_{3}}\left(u^{k+1}\right)\right)\\ \; & \leq L_{G}\alpha_{k}{∥u^{k}-u^{k-1}∥}_{2}+L_{F}{∥u^{k}-u^{k+1}∥}_{2}+\mu {∥u^{k}-u^{k+1}∥}_{2}+\mu \alpha_{k}{∥u^{k}-u^{k-1}∥}_{2}\\ \; & \leq C\left({∥u^{k}-u^{k-1}∥}_{2}+{∥u^{k}-u^{k+1}∥}_{2}\right), \end{array}$$

where $C=\max\left(\left(L_{G}+\mu \right)\bar{\alpha },L_{F}+\mu \right)$. Therefore, it can be seen that F has a finite lower bound, so let $F^{*}≜\inf_{u\in V_{3}}F\left(u\right)$. Consider the following Lemma A1 [40].

**Lemma** **A1.**
*Define $u^{+}=\underset{\chi }{\Pi }\;\left(z-\mu^{-1}\nabla H\left(z\right)\right)$, where $z=u+\alpha \left(u-\bar{u}\right)$, $u,\bar{u}\in \chi$, $0\leq \alpha \leq \bar{\alpha }$, and $H\left(\cdot \right)$ have Lipschitz continuous gradients; the feasible set χ is convex; the gradient step size μ makes the $H\left(\cdot \right)$ satisfy the descent property during the iteration process, that is, $H\left(u^{+}\right)\leq H\left(z\right)+\langle \nabla H\left(z\right),u^{+}-z\rangle +\frac{\mu }{2}{∥u^{+}-z∥}_{2}^{2}$. Then, we obtain $H\left(z\right)-H\left(u^{+}\right)\geq \frac{\mu }{2}{∥u^{+}-u∥}_{2}^{2}-\alpha^{2}{∥u-\bar{u}∥}_{2}^{2}$.*

According to the update rule (21) to (23) of the SoTMM algorithm, we can obtain

(A12) $$\begin{array}{cc} F\left(u^{k}\right)-F\left(u^{k+1}\right) & \overset{\left(a\right)}{\geq }\;G_{\rho }\left(u^{k}\left|u^{k}\right.\right)-G_{\rho }\left(u^{k+1}\left|u^{k}\right.\right)\\ \; & \overset{\left(b\right)}{\geq }\;\frac{\mu }{2}\left({∥u^{k+1}-u^{k}∥}_{2}^{2}-{\bar{\alpha }}^{2}{∥u^{k}-u^{k-1}∥}_{2}^{2}\right), \end{array}$$

where (a) is obtained from $F\left(u\right)=G_{\rho }\left(u\left|u\right.\right)$ and $G_{\rho }\left(u\left|\bar{u}\right.\right)\geq F\left(\bar{u}\right)$; (b) is obtained from Lemma A1 and $\alpha \leq \bar{\alpha }$. Therefore, it can be obtained

(A13) $$\begin{array}{cc} \; & \;\;\;\;\;F\left(u^{0}\right)-F\left(u^{k+1}\right)\\ \; & =\sum_{m=0}^{k}\left(F\left(u^{m}\right)-F\left(u^{m+1}\right)\right)\\ \; & \geq \sum_{m=0}^{k}\frac{\mu }{2}\left({∥u^{m+1}-u^{n}∥}_{2}^{2}-{\bar{\alpha }}^{2}{∥u^{m}-u^{m-1}∥}_{2}^{2}\right)\\ \; & =\sum_{m=0}^{k-1}\frac{\mu }{2}{∥u^{m+1}-u^{m}∥}_{2}^{2}-\sum_{m=-1}^{k-1}\frac{\mu {\bar{\alpha }}^{2}}{2}{∥u^{m+1}-u^{m}∥}_{2}^{2}+\frac{\mu }{2}{∥u^{k+1}-u^{k}∥}_{2}^{2}\\ \; & =\sum_{m=0}^{k-1}\frac{\left(1-{\bar{\alpha }}^{2}\right)\mu }{2}{∥u^{m+1}-u^{m}∥}_{2}^{2}+\frac{\mu }{2}{∥u^{k+1}-u^{k}∥}_{2}^{2}\\ \; & \geq \sum_{m=0}^{k}\frac{\left(1-{\bar{\alpha }}^{2}\right)\mu }{2}{∥u^{m+1}-u^{m}∥}_{2}^{2}. \end{array}$$

We can further rewrite (A13) as

(A14) $$\begin{array}{cc} F\left(u^{0}\right)-F\left(u^{k+1}\right) & \geq \frac{1}{2}\sum_{m=0}^{k}C_{1}{∥u^{m+1}-u^{m}∥}_{2}^{2}+\frac{1}{2}\sum_{m=1}^{k+1}C_{1}{∥u^{m}-u^{m-1}∥}_{2}^{2}\\ \; & \overset{\left(a\right)}{=}\;C_{2}\min_{m=0,\cdots ,k}\;\left({∥u^{m+1}-u^{m}∥}_{2}^{2}+{∥u^{m}-u^{m-1}∥}_{2}^{2}\right), \end{array}$$

where $C_{1}=\left(1-{\bar{\alpha }}^{2}\right)\mu /2\;$, $C_{2}=\left(k+1\right)C_{1}/2\;$. (a) is obtained from $x^{0}=x^{-1}$. Based on the relationship $a+b\leq \sqrt{2\left(a^{2}+b^{2}\right)}$, (A14) can be further rewritten as

(A15) $$\begin{array}{cc} \; & \min_{m=0,\cdots ,k}\;\left({∥u^{m+1}-u^{m}∥}_{2}^{2}+{∥u^{m}-u^{m-1}∥}_{2}^{2}\right)\leq \sqrt{\frac{8}{\left(k+1\right)\left(1-{\bar{\alpha }}^{2}\right)\mu }\left(F\left(u^{0}\right)-F^{*}\right)}. \end{array}$$

Substituting (A15) into (A11), we can obtain

(A16) $$\begin{array}{cc} \min_{m=0,\cdots ,k}\;\mathrm{dist}\left(0,\nabla F\left(u^{m+1}\right)+\partial I_{V_{3}}\left(u^{m+1}\right)\right) & \leq C\min_{m=0,\cdots ,k}\;\left({∥u^{m}-u^{m-1}∥}_{2}+{∥u^{m}-u^{m+1}∥}_{2}\right)\\ \; & \leq C\sqrt{\frac{8}{\left(k+1\right)\left(1-{\bar{\alpha }}^{2}\right)\mu }\left(F\left(u^{0}\right)-F^{*}\right)}. \end{array}$$

In summary, it can be seen that when (A16) is established, $u^{m+1}\in V_{3}$ is the stationary point of the proposed algorithm.
